# Cyclin D1 expression in colorectal cancer is a favorable prognostic factor in men but not in women in a prospective, population-based cohort study

**DOI:** 10.1186/2042-6410-2-10

**Published:** 2011-09-03

**Authors:** Sakarias Wangefjord, Jonas Manjer, Alexander Gaber, Björn Nodin, Jakob Eberhard, Karin Jirström

**Affiliations:** 1 Department of Clinical Sciences, Division of Pathology, Lund University, Skåne University Hospital, 221 85 Lund, Sweden; 2 Department of Clinical Sciences, Division of Surgery, Lund University, Skåne University Hospital, 205 02 Malmö, Sweden; 3 The Malmö Diet and Cancer Study, Lund University, 205 02 Malmö, Sweden; 4 Department of Clinical Sciences, Division of Oncology, Lund University, Skåne University Hospital, 221 85 Lund, Sweden

## Abstract

**Background:**

Although colorectal cancer (CRC) is generally not considered to be a hormone-dependent malignancy, several sex-related differences in incidence, molecular characteristics and survival have been reported. Epidemiological studies have consistently shown that increased exposure to female sex hormones is associated with a lower risk of CRC in women, and cyclin D1, an important downstream effector in estrogen-mediated signaling, is commonly activated in CRC. In this study, we analyzed the prognostic significance of cyclin D1 expression in CRC, with particular reference to sex-related differences, in tumors from a large, prospective, population-based cohort.

**Methods:**

Using tissue microarrays and immunohistochemistry, the fraction and intensity of cyclin D1 expression was evaluated in 527 incident CRC cases from the Malmö Diet and Cancer Study. The χ^2 ^and Spearman's rho (ρ) tests were used for comparison of cyclin D1 expression and relevant clinicopathological characteristics. Kaplan-Meier analysis and Cox proportional hazards modeling were used to assess the effect of cyclin D1 expression on cancer-specific survival (CSS) in univariate and multivariate analysis, adjusted for established prognostic factors.

**Results:**

Cyclin D1 intensity was significantly lower in male compared with female CRC (*P *= 0.018). In the full cohort, cyclin D1 expression was associated with a significantly prolonged CSS (hazard ratio (HR) = 0.69; 95% CI 0.49 to 0.96, *P *= 0.026) but subgroup analysis according to gender revealed a strongly accentuated prognostic effect of cyclin D1 in male CRC (HR = 0.48; 95% CI 0.31 to 0.74, *P *< 0.001), which was in contrast to female CRC, where cyclin D1 was not prognostic (HR = 1.05; 95% CI 0.62 to 1.78, *P *= 0.864) (*P*_interaction _= 0.024). The prognostic value of cyclin D1 was not retained in multivariate analysis, either in the full cohort or in male CRC.

**Conclusions:**

Cyclin D1 expression is strongly associated with prolonged survival in male CRC. These findings not only support an important role for cyclin D1 in colorectal carcinogenesis, but also add further weight to the accumulating evidence that CRC is indeed a hormone-dependent malignancy, for which prognostic and treatment-predictive molecular biomarkers should be evaluated differently in women and men.

## Background

Colorectal cancer (CRC) is one of the most common forms of human cancer worldwide, with approximately 1 million new cases detected every year [1]. Early detection, adequate surgical excision and optimal adjuvant treatment are of crucial importance if a favorable outcome is to be achieved. Currently, tumor stage at diagnosis is the most important prognostic factor in CRC, and although many efforts have been made to find molecular markers to identify high-risk disease and to select patients for adjuvant treatment, none has proven sufficiently good for use in clinical routine.

Several sex-related differences in the incidence [2], survival chemotherapeutic response [4] and certain molecular characteristics [5,6] of CRC have been reported. Furthermore, large-scale population-based studies such as the Women's Health Initiative have shown a significant reduction in both the risk and rate of developing CRC in post-menopausal women treated with combined hormone replacement therapy (HRT) [7], and both pregnancy and the oral contraceptive pill are associated with a reduced CRC risk [8,9]. Taken together, these data suggest that estrogens and/or progestins have a protective effect against colorectal carcinogenesis, although the molecular mechanisms behind these observations are not yet fully understood. The effects of estrogens are mediated by estrogen receptors (ERs), of which two (ERα and ERβ) exist, with ERβ being the predominant ER expressed in CRC [10-12].

Cyclin D1 is an important cell-cycle regulating protein that, together with its binding partners cyclin-dependent kinase (CDK)4 and CDK6, forms active complexes that promote G1- to S-phase progression by phosphorylating and inactivating the retinoblastoma protein [13]. More recent studies have also revealed important CDK-independent functions of cyclin D1 in the regulation of several transcription factors [14], as first shown for the ER [15,16]. Cyclin D1 overexpression is common in CRC, but the findings regarding its prognostic value are conflicting [17-29]. However, the largest study to date, comprising an analysis of 602 tumors from two independent, prospective cohort studies, found an association between cyclin D1 overexpression and a prolonged survival from colon cancer [29].

Cyclin D1 is activated by WNT/β-catenin signaling after mutation of the adenomatous polyposis coli gene (*APC*), an important event in the initiation of colorectal neoplasia [30,31]. WNT/β-catenin signaling is modulated by estrogens in breast cancer [32] and neuronal cells [33], and endogenous estrogens have been found to protect against APC-associated tumor formation in mice, associated with an increase in ERβ and a decrease in ERα expression in the target tissue [34]. Moreover, whereas both ERα and ERβ deficiency have been associated with enhanced intestinal neoplasia in mice carrying *APC *mutations, only ERα deficiency was associated with activation of WNT/β-catenin signaling [35], and functional studies in CRC cells have demonstrated antiproliferative and antitumorigenic effects of ERβ overexpression, despite a functional link to increased cyclin D1 levels [36]. A potential involvement of cyclin D1 in the pathway to CRC, involving mismatch repair, has also been suggested [37], and in the study by Ogino *et al*., an interaction between cyclin D1 expression and microsatellite instability (MSI) status was reported; the presence of either cyclin D1 or high MSI, or both, was associated with a better prognosis [29].

Because cyclin D1 expression is modulated by hormonal activity, we hypothesized that its expression and prognostic effects might differ according to gender in CRC. The aim of this study was therefore to analyze the immunohistochemical expression and prognostic significance of cyclin D1, with particular reference to sex-related differences, in 626 incident cases of CRC in the prospective, population-based cohort Malmö Diet and Cancer Study (MDCS) [38], from which 557 tumors had been assembled in tissue microarrays (TMAs).

## Methods

Ethics approval for the MDCS (reference 51/90) and the present study (reference 530/2008), were obtained from the Ethics Committee at Lund University.

### The Malmö Diet and Cancer Study

The MDCS is a population-based, prospective cohort study with the main aim to examine whether a western diet rich in fat and low in fruit and vegetables increases the risk of certain forms of cancer [38]. Between 1991 and 1996, a total of 28,098 participants (11,063 men (39.4%) and 17.035 women (60.6%)) aged between 44 and 74 years where enrolled (from a background population of 74,138). Follow-up is performed annually by record linkage to national registries for cancer and cause of death.

### Incident colorectal cancer until 31 December 2008

Until the end of follow-up on 31 December 2008, 626 incident cases of CRC had been registered in the study population. Cases were identified from the Swedish Cancer Registry until 31 Dec 2007 and from the Southern Swedish Regional Tumour Registry for the period 1 January to 31 December 2008. All tumors for which slides or paraffin wax tissue blocks were available were histopathologically re-evaluated using hematoxylin and eosin staining. Histopathological, clinical and treatment data were obtained from the clinical- and/or pathology records. Information on vital status and cause of death was obtained from the Swedish Cause of Death Registry until 31 Dec 2009. Follow-up started at date of diagnosis and ended at death, emigration or 31 December 2009, whichever came first.

### Tissue microarray construction

In total, 557 (89.0%) tumors were available and suitable for TMA construction (see Additional File 1). Areas representative of cancer were marked on hematoxylin and eosin-stained slides, and TMAs were constructed as previously described [39]. In brief, two 1.0 mm cores were taken from each tumor and mounted in a new recipient block using a semi-automated arraying device (TMArrayer; Pathology Devices, Westminster, MD, USA).

### Immunohistochemistry and evaluation of cyclin D1 staining

For immunohistochemical analysis, 4 μm TMA sections were automatically pretreated using a pretreatment module (PT-Link; Dako, Glostrup, Denmark) and then stained (Autostainer Plus; Dako) with the monoclonal anti-cyclin D1 antibody DSC-6 (Dako), diluted 1:50. This antibody has been validated and used for staining of formalin-fixed paraffin wax-embedded tissue in several previous studies [40-42].

We recorded the intensity of nuclear cyclin D1 expression (no, weak, moderate or strong), and the proportion of positive tumor cells (0 = 0 to 1%, 1 = 2 to 25%, 2 = 26 to 50%, 3 = 51 to 75%) and 4 = > 75%). For further statistical analyses, cyclin D1 expression was dichotomized into negative (no expression and positive (any expression; fraction and intensity). The staining was evaluated by two independent observers (SW and KJ), who were blinded to the clinical and outcome data. Any scoring differences were discussed in order to reach consensus.

### Statistical analysis

The χ^2 ^and Spearman's ρ tests were used for comparison of cyclin D1 expression and relevant clinicopathological characteristics. Kaplan-Meier analysis and log-rank test were used to illustrate differences in cancer-specific survival (CSS) according to cyclin D1 expression. Cox regression proportional hazards models were used for estimation of hazard ratio (HR) for death from CRC according to cyclin D1 expression in both univariate and multivariate analyses, adjusted for age, gender, TNM stage, differentiation grade and vascular invasion. The interaction between cyclin D1 expression and gender was explored by a Cox model including the interaction variable. All survival analyses were repeated with overall mortality as endpoint and all tests were two-sided. *P *< 0.05 was considered significant. All statistical analyses were performed using SPSS software (version 18; SPSS Inc, Chicago, IL, USA).

## Results

### Distribution of clinicopathological characteristics and cyclin D1 expression in the full cohort and in subgroups according to gender

There was no significant difference in the distribution of clinicopathological characteristics or treatment in subgroups according to gender (Table 1). The distribution of clinicopathological characteristics did not differ between the full cohort (n = 626) and the evaluated cohort (n = 527) (data not shown). There was no sex-related difference in survival for patients with metastatic CRC (Table 1). After antibody optimization and staining, cyclin D1 expression could be evaluated in 527of 557 tumors (94.6%) represented in the TMA. The tissue cores that could not be evaluated either had been lost during immunohistochemical processing or did not contain invasive cancer.

**Table 1:** Patient and tumor characteristics in the evaluated cohort and in subgroups according to gender

	All, n = 527	Female, n = 276 (52.4%)	Male, n = 251 (47.6%)	*P *value^a^
Age				
Mean	70.5	70.6	70.4	0.399
Median	71.4	72	70.9	
Range	49.8 to 85.6	49.8 to 85.2	51.8 to 85.6	
Location				
Colon	323 (61.3)	176 (63.8)	147 (58.6)	0.256
Rectum	190 (36.1)	92 (33.3)	98 (39.0)	
Multiple	12 (2.3)	7 (2.5)	5 (2.0)	
Unknown	2 (0.4)	1 (0.4)	1 (0.4)	
T Stage				
1	46 (8.7)	31 (11.2)	15 (6.0)	0.506
2	63 (12.0)	30 (10.9)	33 (13.1)	
3	319 (60.5)	159 (57.6)	160 (63.7)	
4	78 (14.8)	42 (15.2)	36 (14.3)	
Unknown	21 (4.0)	39 (12.0)	38 (12.7)	
N stage				
0	278 (52.8)	144 (52.2)	134 (53.4)	0.494
1	118 (22.4)	68 (24.6)	50 (19.9)	
2	85 (16.1)	36 (13.0)	49 (19.5)	
Unknown	46 (8.7)	28 (10.1)	18 (7.2)	
M Stage				
0	429 (81.4)	226 (81.9)	203 (80.9)	0.513
1	90 (17.1)	44 (15.9)	46 (18.3)	
Unknown	8 (1.5)	6 (2.2)	2 (0.8)	
Differentiation grade				
High	34 (6.5)	19 (6.9)	15 (6.0)	0.438
Intermediate	367 (69.6)	185 (67.0)	182 (72.5)	
Low	118 (22.4)	67 (24.3)	51 (20.3)	
Unknown	8 (1.5)	5 (1.8)	3 (1.2)	
Vascular invasion				
No	150 (28.5)	81 (29.3)	69 (27.5)	0.635
Yes	156 (29.6)	80 (29.0)	76 (30.3)	
Unknown	222 (41.9)	115 (41.7)	106 (42.2)	
Surgery				
Acute	46 (8.7)	26 (9.4)	20 (8.0)	0.502
Elective	454 (86.1)	233 (84.4)	221 (88.0)	
Unknown	27 (5.1)	17 (6.2)	10 (4.0)	
Neodjuvant treatment				
None	414 (78.6)	216 (83.4)	198 (82.2)	0.291
Short RT	25 (4.7)	10 (3.9)	15 (6.2)	
Long RT	19 (3.6)	8 (3.1)	11 (4.6)	
Chemotherapy + RT	2 (0.4)	2 (0.8)	0 (0.0)	
Chemotherapy	2 (0.4)	1 (0.4)	1 (0.4)	
Unknown	65 (12.3)	39 (14.1)	16 (10.4)	
Adjuvant treatment				
No	294 (55.8)	149 (54.0.8)	145 (57.8)	0.584
FLV/Xeloda	51 (9.7)	25 (9.1)	26 (10.4)	
FLOX/XELOX	19 (3.6)	11 (4.0)	8 (3.2)	
Other	5 (0.9)	4 (1.4)	1 (0.4)	
Curative; M1*	14 (2.7)	6 (2.2)	8 (3.2)	
Palliative	80 (15.2)	39 (14.1)	41 (16.3)	
Unknown	64 (12.1)	42 (15.2)	22 (8.8)	
Follow-up (years)				
Mean	4.7	4,9	4.5	0.399
Median	3.5	3.4	3.4	
Range	0.0 to 17.7	0.0 to 17.7	0.0 to 16.6	
Vital status				
Alive	306 (58.1)	169 (61.2)	137 (54.6)	0.113
Dead	221 (41.9)	107 (38.8)	114 (45.4)	
Dead from CRC	182 (34.5)	90 (32.6)	92 (36.7)	0.313
Follow-up (years); M1 patients				
Mean	1.5	1.3	1.6	0.129
Median	1.1	0.8	1.2	
Range	0.0 to 6.1	0.0 to 5.5	0.0 to 6.1	
Vital status; M1 patients				
Alive	12 (13.3)	7 (15.9)	5 (10.9)	0.484
Dead	78 (86.7)	37 (84.1)	41 (89.1)	
Dead from CRC	78 (86.7)	37 (84.1)	41 (89.1)	0.484
Cyclin D1 fraction				
0 to 1	105 (19.9)	50 (18.1)	55 (21.9)	0.217
02 to 25	195 (37.0)	100 (36.2)	95 (37.8)	
26 to 50	86 (16.3)	48 (17.4)	38 (15.1)	
51 to 75	112 (21.3)	64 (23.2)	48 (19.1)	
> 75	29 (5.5)	14 (5.1)	15 (6.0)	
Cyclin D1 intensity				
Negative	105 (19.9)	50 (18.1)	55 (21.9)	0.018
Weak	181 (34.3)	85 (30.8)	96 (38.2)	
Moderate	176 (33.4)	102 (37.0)	74 (29.5)	
Strong	65 (12.3)	39 (14.1)	26 (10.4)	

**^a^**The *P *values refer to comparisons of male and female tumors, using the Mann-Whitney*U*-test for comparison of medians and the χ^2 ^test for X × 2 tables. P-values for vital status refer to overall and cause-specific death, respectively. The categories marked as 'not done' and 'unknown' were not included in the statistical analysis.

Abbreviations: FLOX = 5-fluorouracil, leucovorin and oxaliplatin, FLV = 5-fluorouracil and leucovorin, RT = radiotherapy, XELOX = Xeloda (capecitabine) and oxaliplatin.

**Figure 1:**
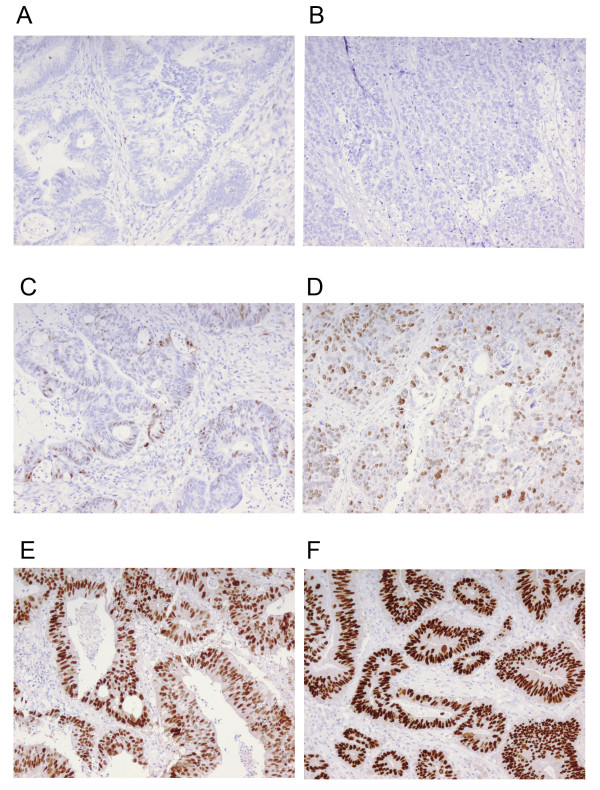
**Immunohistochemical images of cyclin D1 staining in colorectal cancer**. (A,B) Negative staining, (C,D) various fractions of weak to moderate staining, and (D-F) strong cyclin D1 staining. Original magnification × 20.

Representative immunohistochemistry images are shown in Figure 1. Cyclin D1 expression was only rarely seen in inflammatory cells and stromal cells. Of the 527 tumors evaluated, 105 (16.8%) were negative for cyclin D1; in the remaining tumors, cyclin D1 was expressed in various fractions and intensities (Table 1). The intensity but not the fraction of cyclin D1 was significantly lower in male compared with female CRC (*P *= 0.018).

### Association between Cyclin D1 expression and clinicopathological characteristics in the full cohort and the subgroups according to gender

Next, we examined the relationship between cyclin D1 expression (fraction and intensity) and established clinicopathological parameters in all patients, both female and male (Table 2). We found a significant inverse correlation between cyclin D1 fraction (but not intensity) and the N (*R *= -0.014, *P *= 0.012) and M (*R *= -0.091, *P *= 0.039) stages in the full cohort. This association was not evident in women, but in men, both the fraction and intensity of cyclin D1 staining were inversely correlated with N stage (R = -0.134, *P *= 0.041 for fraction and R = -0.153, *P *= 0.020 for intensity) and M stage (R = -0.143, *P *= 0.024 for fraction and R = -0.161, *P *= 0.011 for intensity). In the full cohort, but not in subgroups according to gender, there was a positive association between cyclin D1 fraction (but not intensity) and age (R = 0.101, *P *= 0.020), and an inverse association with T stage (R = -0.105, *P *= 0.018). Cyclin D1 fraction, but not intensity, was also inversely associated with vascular invasion in the full cohort (R = -0.121, *P *= 0.034) and in women (R = -0.175, *P *= 0.026), but not in men.

**Table 2:** Associations between cyclin D1 expression and clinicopathological characteristics in all patients, females and males

	All		Female		Male	
	
	Cyclin D1 fraction	Cyclin D1 intensity	Cyclin D1 fraction	Cyclin D1 intensity	Cyclin D1 fraction	Cyclin D1 intensity
Age						
*R*	0.101	0.064	0.111	0.090	0.083	0.022
*P *value	0.020*	0.142	0.065	0.134	0.189	0.73
n	527	527	276	277	251	251
T stage						
*R*	-0.105	-0-086	-0.105	-0.072	-0.104	-0.098
*P *value	0.018*	0.053	0.09	0.245	0.107	0.128
n	506	506	262	262	244	244
N stage						
*R*	-0.114	-0.079	-0.094	-0.003	-0.134	-0.153
*P *value	0.012*	0.085	0.140	0.962	0.041*	0.020*
n	481	481	248	248	233	233
M stage						
*R*	-0.091	-0.064	-0.039	0.030	-0.143	-0.161
*P *value	0.039*	0.147	0.520	0.628	0.024*	0.011*
n	519	520	270	270	249	249
Differentiation grade						
*R*	-0.051	0.044	-0.095	0.007	-0.004	0.076
*P *value	0.243	0.310	0.116	0.908	0.948	0.232
n	527	527	276	276	251	251
Vascular invasion						
*R*	-0.121	-0.068	-0.175	-0.109	-0.065	-0.018
*P *value	0.034*	0.233	0.026*	0.167	0.435	0.830
n	306	306	161	1611	145	145

Abbreviations: n = number (sample size); R = Spearman's correlation coefficient.

*Significant at the 0.05 level; **Significant at the 0.01 level.

### Association between cyclin D1 expression and survival

**Figure 2:**
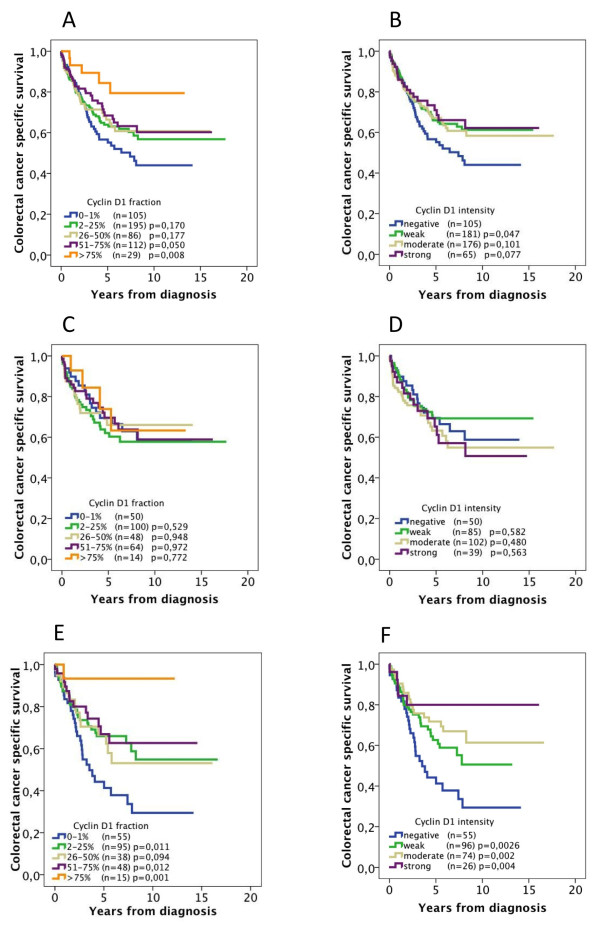
**Association between cyclin D1 expression and survival in all patients and in subgroups according to gender**. Kaplan-Meier analysis of colorectal cancer-specific survival according to cyclin D1 fraction and intensity, respectively, in (A,B) all patients, (C,D) female patients and (E,F) male patients.

The prognostic value of established clinicopathological parameters did not differ between women and men (see Additional File 2). Kaplan-Meier analysis showed that cyclin D1 expression, both fraction and intensity, was associated with a stepwise improvement in CSS in all patients (Figure 2A and 2B). However, subgroup analysis according to gender showed that this association was not significant in female patients (Figure 2C and 2D) but was highly significant, and even accentuated, in male patients (Figure 2E and 2F). These associations were confirmed in univariate Cox regression analysis (Table 3) but did not remain significant in multivariate analysis adjusted for age, gender, TNM, differentiation grade and vascular invasion (Table 3). The results were not significantly altered when vascular invasion or cases with missing information on vascular invasion were excluded from the analysis (data not shown).

**Table 3:** Cox univarate and multivariate proportional hazards analysis of colorectal cancer-specific survival in all patients

Cyclin D1 expression	Univariate				Multivariate			
	
	HR (95%CI)	n (events)	*P*	*P*^a^	HR (95%CI)	n (events)	*P*^b^	*P*^c^
All patients								
Low	1.00	105 (48)			1.00	99 (45)		
High	0.69 (0.49 to 0.96)	422 (134)	0.026	0.024	1.08 (0.75 to 1.58)	377 (113)	0.671	0.429
Female								
Low		1.00	50 (17)			1.00	47 (16)	
High	1.05 (0.62 to 1.78)	226 (73)	0.864		1.35 (0.75 to 2.41)	199 (60)	0.313	
Male								
Low		1.00	55 (31)			1.00	52 (29)	
High	0.48 (0.31 to 0.74)	196 (61)	< 0.001		0.90 (0.53 to 1.53)	178 (53)	0.712	

Abbreviations: HR = hazard ratio.

^a^*P *value from multivariate analysis adjusted for T stage (1 to 2 versus 3 to 4), N stage (0 versus 1 to 2), M stage (0 versus1), age (≤ or ≥ 75 years), differentiation grade (high to intermediate versus low) and vascular invasion (absent, present, missing). Sex was included in the multivariate analysis for all patients.

^b^*P *value for term of interaction by Cox multivariate analysis including gender, the binary covariate cyclin D1 expression and a term of interaction.

^c^*P *value for term of interaction adjusted for T stage (1 to 2 versus 3 to 4), N stage (0 versus 1 to 2), M stage (0 versus1), age (≤ or ≥ 75 years), differentiation grade (high to intermediate versus low) and vascular invasion (absent, present, missing).

**Figure 3:**
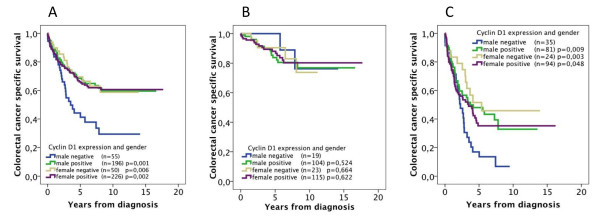
**Kaplan-Meier estimates of colorectal cancer-specific survival according to combinations of gender and positive versus negative cyclin D1 expression**. Colorectal cancer-specific survival in (A) all patients, (B) patients with stage I-II disease and (C) patients with stage III-IV disease. Log-rank *P *values correspond to pairwise comparisons of cyclin D1-negative tumors in male patients with the other strata, respectively.

Cox interaction analysis confirmed a significant interaction between cyclin D1 status and gender (p_interaction _= 0.024), which was not retained when adjusted for conventional prognostic markers (Table 3). Next, we constructed a combined variable of gender and cyclin D1 status, which showed that, in the full cohort, men with cyclin D1-negative tumors had a significantly impaired CSS compared with men with cyclin D1-positive tumors and compared with all women, irrespective of cyclin D1 status (Figure 3A). These associations were not evident in subgroup analysis of patients with stage I-II disease (Figure 3B), but remained significant for patients with stage III-V disease (Figure 3C). In patients with stage III (T1-4, N1-2, M0) disease (n = 126), of whom 65 (51.6%) had received adjuvant chemotherapy and 61 (48.4%) had not, the prognostic value of cyclin D1 was not altered by adjuvant chemotherapy, either in all patients or in subgroup analysis according to gender (data not shown).

The sex-related effect on survival of cyclin D1 expression did not differ between cancers of the colon and rectum (data not shown).

For all analyses, similar associations were seen using overall survival, that is, death from any cause, as endpoint (data not shown).

## Discussion

In this study, we found that cyclin D1 expression was associated with a more favorable outcome from CRC in a large, population-based cohort study, confirming previous findings [29]. However, subgroup analysis according to gender revealed that the prognostic value of cyclin D1 was only evident in male but not female CRC patients, a finding that has, to our knowledge, not been reported previously. It will be of interest for future studies to investigate the molecular basis for this contrasting prognostic significance of cyclin D1 expression in women and men, with particular reference to the influence of sex-hormone levels, anthropometric factors, and genetic and epigenetic modification of steroid receptors.

Although the distribution of conventional clinicopathological and prognostic factors did not differ between female and male CRC patients in this study, the proportion of tumors with strong cyclin D1 intensity was significantly lower in the group of tumors from male patients. This association is interesting, given the protective effect of estrogen against CRC and the important role of cyclin D1 as a mediator of estrogen signaling. Notably, the majority of the female cohort examined in this study was post-menopausal, either having low/no circulating estrogens or taking HRT.

Until further knowledge about the role of cyclin D1 in CRC has been gained, we believe that evaluation of the fraction and intensity of cyclin D1 as separate categories makes sense, not least in light of previous studies in breast cancer, for which the intensity, but not fraction, of cyclin D1 expression was found to influence survival and response to antihormonal therapy [40,43]. However, in our study, there was a similar effect of cyclin D1 on survival for both fraction and intensity, with a stepwise improvement from negative to high expression.

The association between cyclin D1 expression and clinicopathological parameters differed somewhat according to gender, with a significant inverse relationship between cyclin D1 expression and N and M stages in male but not female CRC, indicating that lack of cyclin D1 is associated with a more aggressive phenotype in male patients. However, this interpretation is somewhat dampened by the inverse association we observed between cyclin D1 expression and vascular invasion, another adverse prognostic factor, in female but not male CRC.

In contrast to the findings in the study by Ogino *et al*. [29], cyclin D1 expression did not remain an independent prognostic factor in multivariate analysis in our study, either in the full cohort or in the male group. The association between cyclin D1 expression and more favorable clinicopathological features might in part explain the lack of an independent prognostic value for cyclin D1 in male CRC, although the sex-related prognostic effect of cyclin D1 was evident in patients with stage III-IV disease but not in patients with stage I-II disease. Owing to the relatively small subgroups, these findings should be interpreted with caution, but it is noteworthy that a similar association was found by Ogino *et al*. [29]. The reason for this remains unclear, but it could be speculated that, although they are in a disseminated state, cyclin D1-expressing tumors are still less aggressive than tumors lacking cyclin D1 expression. This assumption is supported by previous studies in breast cancer, which showed an association between low cyclin D1 levels and a more invasive tumor phenotype [42]. Another explanation for the more evident beneficial prognostic effect of cyclin D1 in metastatic disease could be that cyclin D1 expression predicts response to adjuvant chemotherapy. However, we could not find such an effect when the prognostic influence of cyclin D1 expression was analyzed in the subgroup of patients with stage III disease, of whom a relatively large proportion had not received adjuvant chemotherapy.

Because the MDCS is a population-based cohort study, a potential selection bias compared with the general population must be taken into consideration [38]. The denoted frequency of acute surgery was 8.7% (8.3% in the full cohort of 626 cases), which is lower than the usually reported frequency of approximately 25% [44,45]. This is noteworthy as it could reflect a higher awareness of CRC among study participants. However, information on surgery was missing for 5.1% of the patients (8.6% in the full cohort), and lower frequencies have been reported in other studies [46]. Furthermore, the distribution of clinical stages at diagnosis in our study is in line with that expected, with no favoring of less advanced stages.

In this study, we used CRC-specific survival as the primary endpoint. Notably, all associations between cyclin D1 expression and survival were similar when overall survival was used as the endpoint, and because the number of events for cancer-specific and overall survival was identical for patients with metastatic disease, with a median survival of approximately 10.5 months (range 0 to 72), the use of CSS should be a reasonable surrogate for cancer-specific outcome. In future studies, the effect of cyclin D1 expression on recurrence-free survival should also be assessed, preferably in cohorts in which this information has been recorded prospectively.

## Conclusions

The results from this large cohort study show that tumor-specific cyclin D1 expression is strongly associated with a prolonged survival from CRC in men but not women. These findings not only suggest an important role for cyclin D1 in colorectal carcinogenesis and progression, but also add support to the accumulating evidence that sex hormones are relevant to the development of CRC, and that prognostic and treatment-predictive molecular biomarkers should be evaluated differently in women and men.

## List of abbreviations

APC: adenomatous polyposis coli; CDK: cyclin-dependent kinase; CRC: colorectal cancer; CSS: cancer-specific survival; ER: estrogen receptor; HRT: hormone replacement therapy; MDCS: Malmö Diet and Cancer Study MSI: microsatellite instability.

## Competing interests

The authors declare that they have no competing interests.

## Authors' contributions

SW participated in the data collection, performed the statistical analyses and drafted the manuscript. AG assisted with the data collection and statistical analyses. BN assisted with the data collection and constructed the tissue microarrays. JM and JE assisted with the data collection, and helped to draft the manuscript. KJ conceived of the study, performed the histopathological re-evaluation, assisted with the data collection and helped to draft the manuscript. All authors read and approved the final manuscript.

## Supplementary Material

Additional file 1 **Figure S1**. Flowchart describing the availability of tumors and for tissue microarray construction. Click here for file

Additional file 2 **Table S1**. Prognostic value in Cox univariate analysis for established clinicopathological parameters in all patients, female patient and male patients, respectively. Click here for file

## References

[B1] Parkin DM Bray F Ferlay J Pisani P Global cancer statistics, 2002 CA Cancer J Clin 2005 55 74 108 10.3322/canjclin.55.2.74 15761078

[B2] DeCosse JJ Ngoi SS Jacobson JS Cennerazzo WJ Gender and colorectal cancer Eur J Cancer Prev 1993 2 105 115 10.1097/00008469-199303000-00003 8461861

[B3] Press OA Zhang W Gordon MA Yang D Lurje G Iqbal S El-Khoueiry A Lenz HJ Gender-related survival differences associated with EGFR polymorphisms in metastatic colon cancer Cancer Res 2008 68 3037 3042 10.1158/0008-5472.CAN-07-2718 18413774

[B4] Elsaleh H Joseph D Grieu F Zeps N Spry N Iacopetta B Association of tumour site and sex with survival benefit from adjuvant chemotherapy in colorectal cancer Lancet 2000 355 1745 1750 10.1016/S0140-6736(00)02261-3 10832824

[B5] Slattery ML Potter JD Curtin K Edwards S Ma KN Anderson K Schaffer D Samowitz WS Estrogens reduce and withdrawal of estrogens increase risk of microsatellite instability-positive colon cancer Cancer Res 2001 61 126 130 11196149

[B6] Breivik J Lothe RA Meling GI Rognum TO Borresen-Dale AL Gaudernack G Different genetic pathways to proximal and distal colorectal cancer influenced by sex-related factors Int J Cancer 1997 74 664 669 10.1002/(SICI)1097-0215(19971219)74:6<664::AID-IJC18>3.0.CO;2-5 9421366

[B7] Rossouw JE Anderson GL Prentice RL LaCroix AZ Kooperberg C Stefanick ML Jackson RD Beresford SA Howard BV Johnson KC Risks and benefits of estrogen plus progestin in healthy postmenopausal women: principal results From the Women's Health Initiative randomized controlled trial Jama 2002 288 321 333 10.1001/jama.288.3.321 12117397

[B8] La Vecchia C Franceschi S Reproductive factors and colorectal cancer Cancer Causes Control 1991 2 193 200 10.1007/BF00056213 1873449

[B9] La Vecchia C Bosetti C Oral contraceptives and neoplasms other than breast and female genital tract Eur J Cancer Prev 2009 18 407 411 10.1097/CEJ.0b013e32832caaca 19609214

[B10] Campbell-Thompson M Lynch IJ Bhardwaj B Expression of estrogen receptor (ER) subtypes and ERbeta isoforms in colon cancer Cancer Res 2001 61 632 640 11212261

[B11] Wong NA Malcomson RD Jodrell DI Groome NP Harrison DJ Saunders PT ERbeta isoform expression in colorectal carcinoma: an in vivo and in vitro study of clinicopathological and molecular correlates J Pathol 2005 207 53 60 10.1002/path.1807 15954165

[B12] Xie LQ Yu JP Luo HS Expression of estrogen receptor beta in human colorectal cancer World J Gastroenterol 2004 10 214 217 14716825 10.3748/wjg.v10.i2.214 PMC4717006

[B13] Alao JP The regulation of cyclin D1 degradation: roles in cancer development and the potential for therapeutic invention Mol Cancer 2007 6 24 17407548 10.1186/1476-4598-6-24 PMC1851974

[B14] Coqueret O Linking cyclins to transcriptional control Gene 2002 299 35 55 10.1016/S0378-1119(02)01055-7 12459251

[B15] Zwijsen RM Wientjens E Klompmaker R van der Sman J Bernards R Michalides RJ CDK-independent activation of estrogen receptor by cyclin D1 Cell 1997 88 405 415 10.1016/S0092-8674(00)81879-6 9039267

[B16] Neuman E Ladha MH Lin N Upton TM Miller SJ DiRenzo J Pestell RG Hinds PW Dowdy SF Brown M Ewen ME Cyclin D1 stimulation of estrogen receptor transcriptional activity independent of cdk4 Mol Cell Biol 1997 17 5338 5347 9271411 10.1128/mcb.17.9.5338 PMC232384

[B17] Maeda K Chung Y Kang S Ogawa M Onoda N Nishiguchi Y Ikehara T Nakata B Okuno M Sowa M Cyclin D1 overexpression and prognosis in colorectal adenocarcinoma Oncology 1998 55 145 151 10.1159/000011849 9499189

[B18] Handa K Yamakawa M Takeda H Kimura S Takahashi T Expression of cell cycle markers in colorectal carcinoma: superiority of cyclin A as an indicator of poor prognosis Int J Cancer 1999 84 225 233 10.1002/(SICI)1097-0215(19990621)84:3<225::AID-IJC5>3.0.CO;2-A 10371338

[B19] Palmqvist R Stenling R Oberg A Landberg G Expression of cyclin D1 and retinoblastoma protein in colorectal cancer Eur J Cancer 1998 34 1575 1581 10.1016/S0959-8049(98)00162-2 9893631

[B20] Bukholm IK Nesland JM Protein expression of p53, p21 (WAF1/CIP1), bcl-2, Bax, cyclin D1 and pRb in human colon carcinomas Virchows Arch 2000 436 224 228 10.1007/s004280050034 10782880

[B21] Holland TA Elder J McCloud JM Hall C Deakin M Fryer AA Elder JB Hoban PR Subcellular localisation of cyclin D1 protein in colorectal tumours is associated with p21(WAF1/CIP1) expression and correlates with patient survival Int J Cancer 2001 95 302 306 10.1002/1097-0215(20010920)95:5<302::AID-IJC1052>3.0.CO;2-# 11494229

[B22] McKay JA Douglas JJ Ross VG Curran S Loane JF Ahmed FY Cassidy J McLeod HL Murray GI Analysis of key cell-cycle checkpoint proteins in colorectal tumours J Pathol 2002 196 386 393 10.1002/path.1053 11920733

[B23] Bahnassy AA Zekri AR El-Houssini S El-Shehaby AM Mahmoud MR Abdallah S El-Serafi M Cyclin A and cyclin D1 as significant prognostic markers in colorectal cancer patients BMC Gastroenterol 2004 4 22 10.1186/1471-230X-4-22 15385053 PMC524166

[B24] Bondi J Bukholm G Nesland JM Bukholm IR Expression of non-membranous beta-catenin and gamma-catenin, c-Myc and cyclin D1 in relation to patient outcome in human colon adenocarcinomas APMIS 2004 112 49 56 10.1111/j.1600-0463.2004.apm1120109.x 14961975

[B25] Bondi J Husdal A Bukholm G Nesland JM Bakka A Bukholm IR Expression and gene amplification of primary (A, B1, D1, D3, and E) and secondary (C and H) cyclins in colon adenocarcinomas and correlation with patient outcome J Clin Pathol 2005 58 509 514 10.1136/jcp.2004.020347 15858123 PMC1770669

[B26] Knosel T Emde A Schluns K Chen Y Jurchott K Krause M Dietel M Petersen I Immunoprofiles of 11 biomarkers using tissue microarrays identify prognostic subgroups in colorectal cancer Neoplasia 2005 7 741 747 10.1593/neo.05178 16207476 PMC1501883

[B27] Kouraklis G Theocharis S Vamvakas P Vagianos C Glinavou A Giaginis C Sioka C Cyclin D1 and Rb protein expression and their correlation with prognosis in patients with colon cancer World J Surg Oncol 2006 4 5 10.1186/1477-7819-4-5 16426443 PMC1360071

[B28] Lyall MS Dundas SR Curran S Murray GI Profiling markers of prognosis in colorectal cancer Clin Cancer Res 2006 12 1184 1191 10.1158/1078-0432.CCR-05-1864 16489072

[B29] Ogino S Nosho K Irahara N Kure S Shima K Baba Y Toyoda S Chen L Giovannucci EL Meyerhardt JA Fuchs CS A cohort study of cyclin D1 expression and prognosis in 602 colon cancer cases Clin Cancer Res 2009 15 4431 4438 10.1158/1078-0432.CCR-08-3330 19549773 PMC2921858

[B30] Shtutman M Zhurinsky J Simcha I Albanese C D'Amico M Pestell R Ben-Ze'ev A The cyclin D1 gene is a target of the beta-catenin/LEF-1 pathway Proc Natl Acad Sci USA 1999 96 5522 5527 10.1073/pnas.96.10.5522 10318916 PMC21892

[B31] Tetsu O McCormick F Beta-catenin regulates expression of cyclin D1 in colon carcinoma cells Nature 1999 398 422 426 10.1038/18884 10201372

[B32] Hu ZZ Kagan BL Ariazi EA Rosenthal DS Zhang L Li JV Huang H Wu C Jordan VC Riegel AT Wellstein A Proteomic analysis of pathways involved in estrogen-induced growth and apoptosis of breast cancer cells PLoS ONE 6 e20410 10.1371/journal.pone.0020410 PMC3124472 21738574

[B33] Wandosell F Varea O Arevalo MA Garcia-Segura LM Oestradiol regulates beta-catenin-mediated transcription in neurones J Neuroendocrinol10.1111/j.1365-2826.2011.02186.x 21722217

[B34] Javid SH Moran AE Carothers AM Redston M Bertagnolli MM Modulation of tumor formation and intestinal cell migration by estrogens in the Apc(Min/+) mouse model of colorectal cancer Carcinogenesis 2005 26 587 595 15579483 10.1093/carcin/bgh346

[B35] Cleveland AG Oikarinen SI Bynote KK Marttinen M Rafter JJ Gustafsson JA Roy SK Pitot HC Korach KS Lubahn DB Disruption of estrogen receptor signaling enhances intestinal neoplasia in Apc(Min/+) mice Carcinogenesis 2009 30 1581 1590 10.1093/carcin/bgp132 19520794 PMC2736300

[B36] Hartman J Edvardsson K Lindberg K Zhao C Williams C Strom A Gustafsson JA Tumor repressive functions of estrogen receptor beta in SW480 colon cancer cells Cancer Res 2009 69 6100 6106 19602591 10.1158/0008-5472.CAN-09-0506

[B37] Nosho K Kawasaki T Chan AT Ohnishi M Suemoto Y Kirkner GJ Fuchs CS Ogino S Cyclin D1 is frequently overexpressed in microsatellite unstable colorectal cancer, independent of CpG island methylator phenotype Histopathology 2008 53 588 598 10.1111/j.1365-2559.2008.03161.x 18983468 PMC2719983

[B38] Berglund G Elmstahl S Janzon L Larsson SA The Malmo Diet and Cancer Study. Design and feasibility J Intern Med 1993 233 45 51 10.1111/j.1365-2796.1993.tb00647.x 8429286

[B39] Kononen J Bubendorf L Kallioniemi A Barlund M Schraml P Leighton S Torhorst J Mihatsch MJ Sauter G Kallioniemi OP Tissue microarrays for high-throughput molecular profiling of tumor specimens Nat Med 1998 4 844 847 10.1038/nm0798-844 9662379

[B40] Jirstrom K Stendahl M Ryden L Kronblad A Bendahl PO Stal O Landberg G Adverse effect of adjuvant tamoxifen in premenopausal breast cancer with cyclin D1 gene amplification Cancer Res 2005 65 8009 8016 16140974 10.1158/0008-5472.CAN-05-0746

[B41] Borgquist S Wirfalt E Jirstrom K Anagnostaki L Gullberg B Berglund G Manjer J Landberg G Diet and body constitution in relation to subgroups of breast cancer defined by tumour grade, proliferation and key cell cycle regulators Breast Cancer Res 2007 9 R11 10.1186/bcr1644 17254341 PMC1851395

[B42] Lehn S Tobin NP Berglund P Nilsson K Sims AH Jirstrom K Harkonen P Lamb R Landberg G Down-regulation of the oncogene cyclin D1 increases migratory capacity in breast cancer and is linked to unfavorable prognostic features Am J Pathol 177 2886 2897 10.2353/ajpath.2010.100303 PMC2993304 20971731

[B43] Stendahl M Kronblad A Ryden L Emdin S Bengtsson NO Landberg G Cyclin D1 overexpression is a negative predictive factor for tamoxifen response in postmenopausal breast cancer patients Br J Cancer 2004 90 1942 1948 10.1038/sj.bjc.6601831 15138475 PMC2409465

[B44] Scott NA Jeacock J Kingston RD Risk factors in patients presenting as an emergency with colorectal cancer Br J Surg 1995 82 321 323 10.1002/bjs.1800820311 7795995

[B45] Sjo OH Larsen S Lunde OC Nesbakken A Short term outcome after emergency and elective surgery for colon cancer Colorectal Dis 2009 11 733 739 10.1111/j.1463-1318.2008.01613.x 18624817

[B46] Pavlidis TE Marakis G Ballas K Rafailidis S Psarras K Pissas D Sakantamis AK Does emergency surgery affect resectability of colorectal cancer? Acta Chir Belg 2008 108 219 225 18557147 10.1080/00015458.2008.11680207

