# Supplementary figures and images for: Cyclin D1 expression in colorectal cancer is a favorable prognostic factor in men but not in women in a prospective, population-based cohort study
Source: Biol Sex Differ. 2011 Sep 3;2:10. doi: 10.1186/2042-6410-2-10 (PMC3179695; doi:10.1186/2042-6410-2-10)

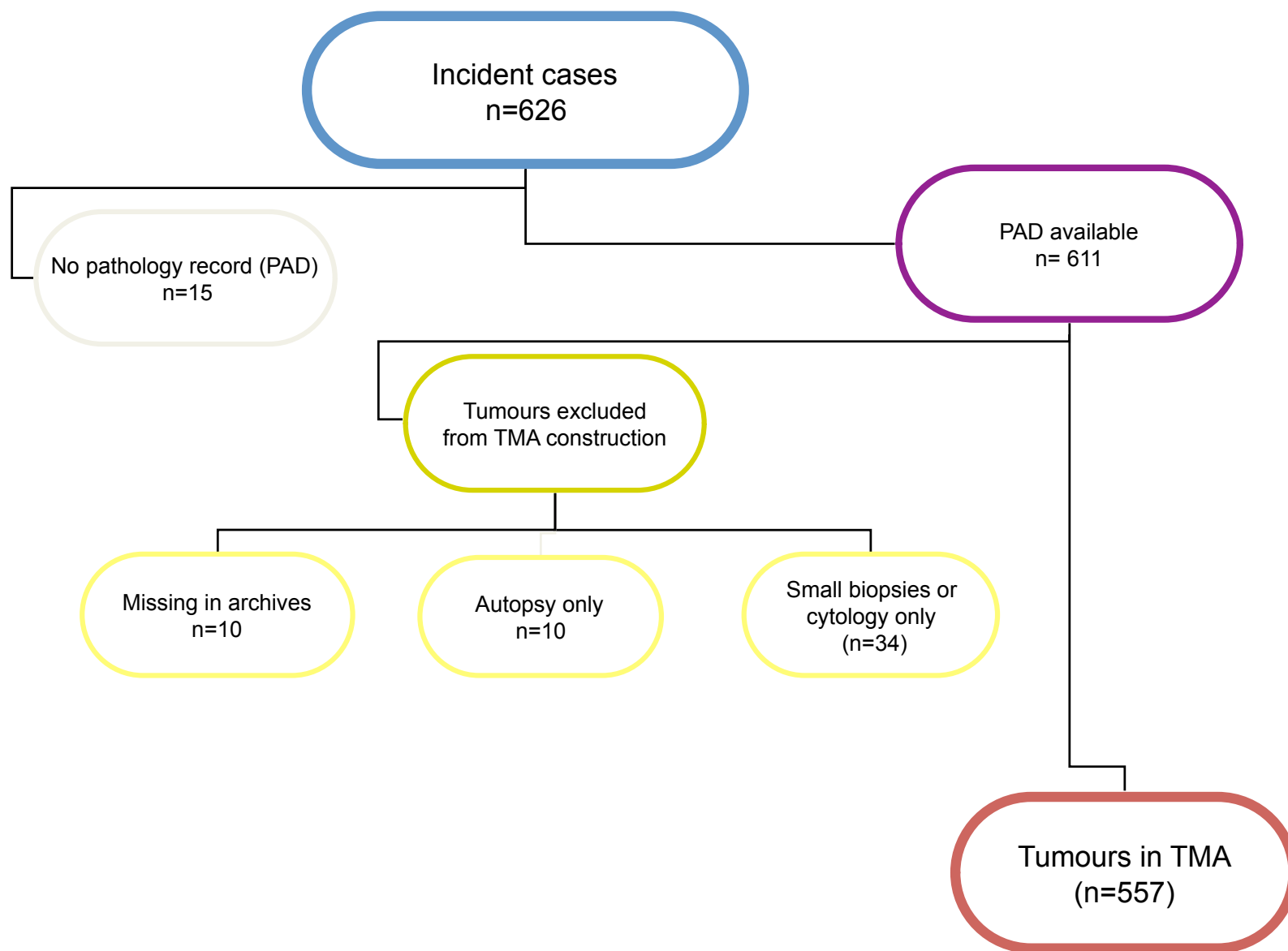

Supplement: Additional file 1 — Figure S1. Flowchart describing the availability of tumors and for tissue microarray construction. [file 2042-6410-2-10-S1.PDF]
