# Supplementary material for: Cyclin D1 expression in colorectal cancer is a favorable prognostic factor in men but not in women in a prospective, population-based cohort study
Source: Biol Sex Differ. 2011 Sep 3;2:10. doi: 10.1186/2042-6410-2-10 (PMC3179695; doi:10.1186/2042-6410-2-10)
Supplement: Additional file 2 — Table S1. Prognostic value in Cox univariate analysis for established clinicopathological parameters in all patients, female patient and male patients, respectively. [file 2042-6410-2-10-S2.DOC]

**Additional Table 1. Prognostic value in Cox univariate analysis for established clinicopathological parameters in all patients, females and males, respectively.**

|  | **All** |  |  |  | **Female** |  |  |  | **Male** |  |  |
| --- | --- | --- | --- | --- | --- | --- | --- | --- | --- | --- | --- |
|  | HR (95% CI) | p | n(events) |  | HR (95% CI) | p | n(events) |  | HR (95% CI) | p | n(events) |
|  |  |  |  |  |  |  |  |  |  |  |  |
| **Age** |  |  |  |  |  |  |  |  |  |  |  |
| <=75 | 1.00 |  | 428(163) |  | 1.00 |  | 222(83) |  | 1.00 |  | 206(80) |
| >75 | 1,34 (1,01-1,77) | 0.044 | 198(72) |  | 1,29 (0,87-1,92) | 0.198 | 104(37) |  | 1,38 (0,92-2,07) | 0.114 | 94(35) |
|  |  |  |  |  |  |  |  |  |  |  |  |
| **T-stage** |  |  |  |  |  |  |  |  |  |  |  |
| 1+2 | 1.00 |  | 116(12) |  | 1.00 |  | 63(3) |  | 1.00 |  | 53(9) |
| 3 | 3,22(1,77-5,84) | <0.001 | 346(109) |  | 6,39(1,99-20,45) |  | 174(52) |  | 2,16(1,07-4,37) | 0,032 | 172(57) |
| 4 | 11,68(6,27-21,75) | <0.001 | 87(60) |  | 24,85(7,64-80,79) |  | 50(36) |  | 7,19(3,33-15,56) | <0.001 | 37(24) |
|  |  |  |  |  |  |  |  |  |  |  |  |
| **N-stage** |  |  |  |  |  |  |  |  |  |  |  |
| 0 | 1.00 |  | 305(55) |  | 1.00 |  | 162(27) |  | 1.00 |  | 143(28) |
| 1 | 3,13 (2,16-4,52) | <0,001 | 130(58) |  | 3,37 (2,12-5,64) | <0,001 | 75(32) |  | 2,89 (1,69-4,94) | <0,001 | 55(26) |
| 2 | 7,54 (5,24-10,87) | <0,001 | 90(65) |  | 8,02 (4,70-13,68) | <0,001 | 37(28) |  | 7,03 (4,25-11,61) | <0,001 | 53(37) |
|  |  |  |  |  |  |  |  |  |  |  |  |
| **M-stage** |  |  |  |  |  |  |  |  |  |  |  |
| 0 | 1.00 |  | 475(111) |  | 1.00 |  | 250(56) |  | 1.00 |  | 225(55) |
| 1 | 12,49 (9,31-16,76) | <0,001 | 125(111) |  | 13,32 (8,80-20,16) | <0,001 | 62(55) |  | 11,28 (7,45-17,08) | <0,001 | 63(56) |
|  |  |  |  |  |  |  |  |  |  |  |  |
| **Differentiation grade** |  |  |  |  |  |  |  |  |  |  |  |
| high + intermediate | 1.00 |  | 448(133) |  | 1.00 |  | 229(63) |  | 1.00 |  | 219(70) |
| low | 2,33 (1,75-3,11) | <0,001 | 130(71) |  | 2,54 (1,71-3,76) | <0,001 | 75(41) |  | 2,11 (1,38-3,24) | 0.001 | 55(30) |
|  |  |  |  |  |  |  |  |  |  |  |  |
| **Vascular invasion** |  |  |  |  |  |  |  |  |  |  |  |
| no | 1.00 |  | 158(24) |  | 1.00 |  | 86(12) |  | 1.00 |  | 72(12) |
| yes | 4,19 (2,68-6,55) | <0,001 | 170(96) |  | 4,24 (2,26-7,98) | <0,001 | 90(49) |  | 4,17 (2,21-7,87) | <0,001 | 80(47) |
|  |  |  |  |  |  |  |  |  |  |  |  |
|  |  |  |  |  |  |  |  |  |  |  |  |
